# Surface Analysis of TMCTS-Based SiOC(H) Low-*k* Dielectrics in Post-Etch Strip of ACL Hardmask

**DOI:** 10.3390/ma14051144

**Published:** 2021-02-28

**Authors:** Min Kyu Park, Wan Soo Song, Min Ho Kim, Sang Jeen Hong

**Affiliations:** Department of Electronics Engineering, Myongji University, Yongin 17058, Korea; pmg1007@hanmail.net (M.K.P.); wansoo2603@naver.com (W.S.S.); frofaste@naver.com (M.H.K.)

**Keywords:** surface analysis, strip process, OES, amorphous carbon, SiOC(H), hardmask, low-*k*

## Abstract

The miniaturization of devices requires the introduction of a high aspect ratio through patterning in the Damascene copper interconnect process. The high aspect ratio etch process employs hardmasks, such as amorphous carbon, that can withstand high-powered plasma exposure. When an etch hardmask is removed after patterning, the properties of the underlying film can be altered by the effect of plasma exposure during the strip process. In this study, surface properties of SiOC(H) are investigated after an amorphous carbon strip with O_2_/Ar plasma. Since the low-k film of SiOC(H) structure shows characteristics according to the Si-O internal bonding structure, the Si-O bonding ratio of the ring, network and cage structure was analyzed through Fourier-transform infrared (FT-IR) analysis to measure changes in thin film properties. X-ray photoelectron spectroscopy (XPS) was also used to add reliability to the SiOC(H) film structure. In addition, the end point of the strip process was obtained using an optical emission spectroscopy sensor and variations in thin film characteristics over the plasma exposure time were analyzed. These results revealed the structural modification of the SiCO(H) thin film in the post-etch strip of the amorphous carbon layer (ACL) hardmask.

## 1. Introduction

Device miniaturization over the last several decades has improved semiconductor technology performance and reduced power requirements. For example, graphics processing units designed for accelerated computing outperform conventional single-thread central processors [1]. However, delay caused by the interconnects in highly integrated devices for rapid computing is a significant problem. Cu and low-*k* dielectrics were adopted in the late 1990s to mitigate interconnect delay. Efforts to reduce the dielectric constants of interlayer dielectric materials by introducing pores are ongoing [2]. The k-value of a dielectric film with weakly polar bonds and a porous structure can be reduced by annealing it at an elevated temperature, but the thermal budget for back-end-of-line (BEOL) interconnects remains an obstacle to process integration.

Organosilicate glass (OSG), also known as carbon-doped silicon glass, fluorinated silicon glass, and spin-on dielectrics are conventional low-*k* dielectric materials. However, the flexibility of gas-phase processing makes plasma-enhanced chemical vapor deposition (PECVD) the preferred fabrication method for low-k dielectric films consisting of dual-phase SiOCH–CH_x_ materials. Skeletal precursors, such as tetramethylcyclotetrasiloxane (TMCTS), have suitable electrical and mechanical properties for porous low-*k* dielectrics [3,4], but plasma-induced damage to low-k interlayer dielectrics (ILDs) designed as interconnects for semiconducting logic devices should be minimized [5]. Plasma-induced damage to porous SiOC(H) low-*k* dielectrics following the etching process has been reported. The application of hard SiON/TiN/SiON masks and pulsed plasma etching have been reported to reduce damage to porous SiOC(H) thin films [6,7]. Etches introduced during the BEOL process are extremely narrow (<45 nm), so if continuous wave plasma is used for etching, ultraviolet (UV), vacuum ultraviolet (VUV), and high-energy ion irradiation seriously damage the sidewalls. Pulsed radiofrequency (RF) plasma creates more isotropic etches in porous SiOC(H) and causes less damage.

The dual damascene process used to prepare copper interconnects can generate vias with high aspect ratios, and the use of hardmasks has become routine. Torazawa et al. integrated extremely low-k dielectric films and used a hard metal masking process to form copper interconnects. The SiOC(H) films were deposited via PECVD, then irradiated under UV light to remove the porogens [8]. Lai et al. used amorphous carbon (*a*-C) to enhance hard masking and lithography capability, and they found that C_2_H_2_-based *a*-C provided better sidewall step coverage than conventional C_3_H_3_-based *a*-C [9]. However, applying an amorphous carbon layer (ACL) as a hardmask interferes with overlay alignment, so efforts to develop hard masking schemes are ongoing [10,11].

A variety of low-*k* dielectric deposition and etching processes exist, and hardmasks can be employed to create patterns with high aspect ratios. However, studies on removing hardmasks from low-*k* dielectrics after etching have been limited. The dielectric constant of a low-k dielectric depends on the structural characteristics of the material, and the dielectric constant of a deposited low-k dielectric may differ from that of pristine material. Many factors can affect the dielectric constants of low-k dielectrics, such as the hardmask deposition temperature, UV/VUV radiation emitted by the plasma during deposition, ion bombardment, and surface reactions that occur during post-etch stripping. Depending on the type of hardmask used and the structural properties of a material, gases generated after stripping may contain fluorinated species that cause undesired multi-step chemical reactions [12]. In this research, we prepared TMCTS-based low-k dielectric films via PECVD and deposited ACLs to serve as hardmasks. We then stripped off the hardmasks and analyzed the surfaces of the films to investigate changes in their structures and surface chemical compositions. We postulated that the ACL hardmask deposition process and post-etch stripping could alter the characteristics of the pristine material.

## 2. Experimental

**Figure 1:**
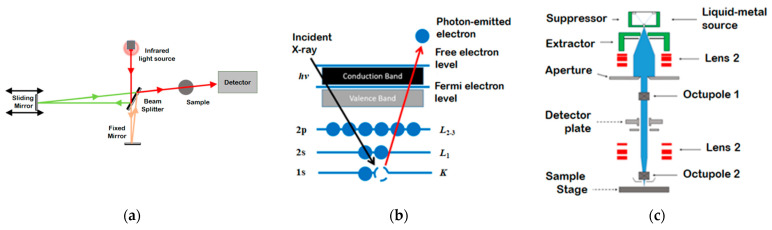
Illustrations of the analytical tools used to characterize the film surfaces: (**a**) Fourier-transform infrared (FT-IR), (**b**) X-ray photoelectron spectroscopy (XPS), and (**c**) field emission scanning electron microscope (FE-SEM).

Fourier-transform infrared (FT-IR) spectroscopy, X-ray photoelectron spectroscopy (XPS), and hardness measurements were performed to identify changes in the surface properties of the films. After each process integration step, we analyzed the chemical composition and hardness of each low-*k* thin film, as well as processes that occurred on the surface. Schematic diagrams of the XPS process, an FT-IR spectrometer, and a field emission scanning electron microscope (FE-SEM) are shown in Figure 1. FT-IR was performed to analyze Si–O bonds, which provided information about structural changes on the surfaces of the films due to plasma ion bombardment. The intensity of the peak associated with Si–CH_3_ bonds was also investigated to determine the internal carbon contents of the processed low-*k* films. XPS was conducted to investigate the atomic distributions and chemical compositions on the surfaces of the films. We investigated surface conditions after depositing the ACL hardmasks on the TMCTS-based SiOC(H) low-*k* dielectric films; after exposing the ACL hardmasks to plasma; and after plasma stripping of the ACL hardmasks for an extended period of time to induce surface damage. Structural changes in the thin films were also investigated to identify the binding state of Si after fitting the results.

Low-*k* dielectrics tend to be softer and more porous than conventional dielectrics. We wondered whether plasma ion bombardment would modify surface hardness, which would indicate that the plasma physically damaged the low-*k* dielectrics. We performed surface indentation tests to investigate the hardness and modulus of each thin film. Hardness can vary due to differences in the physical and chemical characteristics of films, such as their chemical bonding structures and grain sizes. We also performed endpoint detection (EPD) via optical emission spectroscopy (OES) to assess the suitability of the ACL hard masking conditions. Changes in the intensities of the optical emission peaks corresponding to CO and O were monitored to identify changes in the chemical species present in the plasma. Finally, we collected cross-sectional FE-SEM images and compared the ACL stripping process times and thin film thicknesses according to the applied RF power.

**Figure 2:**
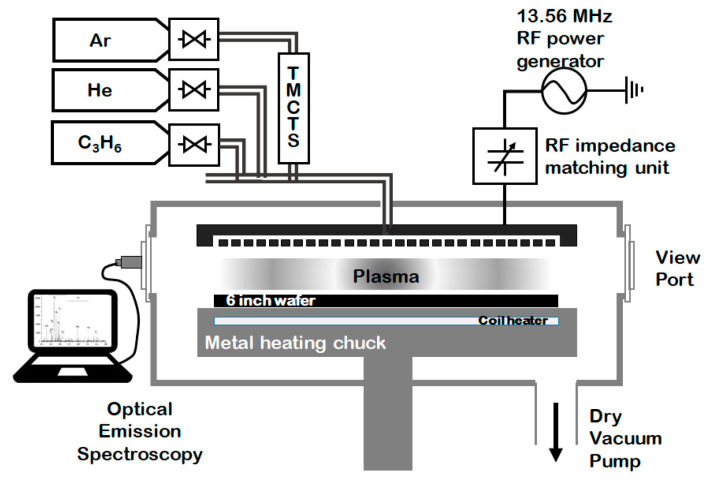
A schematic diagram of the plasma-enhanced chemical vapor deposition (PECVD) apparatus used to deposit the low-*k* dielectrics and the amorphous carbon layer (ACL) hardmasks.

The dielectric films were deposited on six-inch test grade <100> silicon wafers via capacitively coupled plasma (CCP) PECVD at an RF power of 13.56 MHz. SiOC(H) low-*k* films were deposited on the tetramethylcyclotetrasiloxane (TMCTS, Si_4_C_4_H_16_O_4_) precursor (DNF, Daejeon, Korea) in a multipurpose six-inch PECVD chamber. The TMCTS precursor was passed through a heated 10 mL bubbler at 50 °C using He as the carrier gas and O_2_ as the reactive gas, which were mixed at the shower head. A schematic diagram of the PECVD system is presented in Figure 2.

Once the low-*k* dielectrics were deposited, ACL hardmasks were directly deposited onto the films using the same PECVD apparatus. The chamber was flushed with NF_3_ for an extended time between the deposition steps to prevent cross-contamination. The ACL films were deposited in a mixture of C_3_H_6_ and He gases. The characteristics of the TMCTS-based low-*k* films, including their structures, were sensitive to the post-deposition annealing temperature. Annealing had to be performed at a specific temperature for a certain amount of time to achieve the desired material composition. The annealing temperature (400 °C) and time were selected based on the subsequent ACL deposition process, which was also performed at 400 °C, to limit physical changes in the low-*k* dielectric and obtain thin films with characteristics consistent with high-temperature deposition [13]. The properties of hydrogenated amorphous carbon were evaluated by determining the *sp*^3^ and *sp*^2^ orbital ratio and the amount of hydrogen in the films. A hydrogen content of 20–40% and a low *sp*^3^ content were indicative of a C–C *sp*^3^ bond structure, like that of diamond, which afforded good resistance to plasma ion bombardment. However, attempting to increase the deposition rate by raising the process temperature to over 400 °C reduced the hydrogen concentration and transformed the *sp^3^* network into a *sp*^2^ network, which compromised etching resistance.

**Figure 3:**
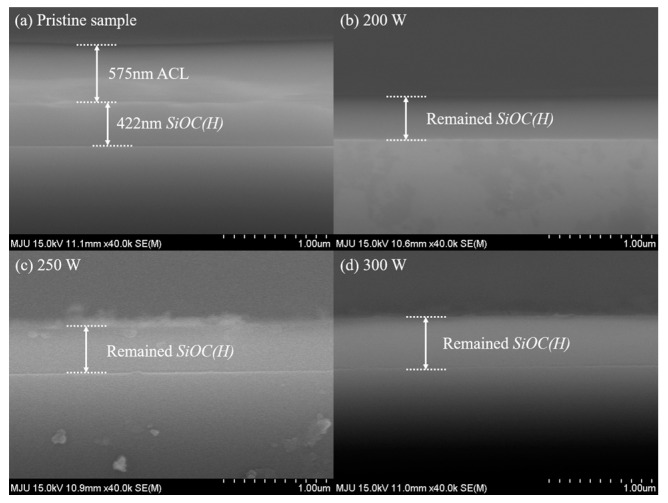
SEM images of a pristine sample and over-stripped samples.

Subsequent plasma stripping to remove the deposited ACL hardmasks was conducted using the CCP system. The stripping system consisted of a 13.56 MHz power source that supplied power through the top of the electrode. The main process control knobs were used to regulate the RF source power, pressure, chuck temperature, and gas flow. Generating photolithography patterns on the ACLs would mimic the actual semiconductor integration process. However, we etched the backs of the ACL hardmasks to investigate changes in the characteristics of the underlying material after plasma stripping. The 400 nm thick ACL hardmasks were blanket-etched with 50 sccms of an O_2_/Ar gas mixture for 300 s at a base pressure of 300 mTorr. RF source powers of 200, 250, and 300 W were applied during the ACL stripping process to investigate the effect of RF power on the bond structures of the low-*k* thin films. FE-SEM images of pristine and over-etched samples stripped at 200, 250, and 300 W are shown in Figure 3.

To establish an appropriate stripping endpoint, film etching rates based on processing time were determined by analyzing the FE-SEM images and the OES data. The total areas of the C=C and Si–O peaks and their intensities after stripping under each set of conditions were compared to evaluate the characteristics of the ACL hardmasks. Decreases in the intensity of the CO peak appearing at 288 nm over time were also monitored. The endpoint was measured in the range from 250 to 260 s while applying a source power of 200 W, and the endpoints became shorter as the source power increased. Increasing the plasma exposure time and source power were expected to significantly alter the properties of the thin films.

## 3. Results

### 3.1. Endpoint Detection (EPD)

**Figure 4:**
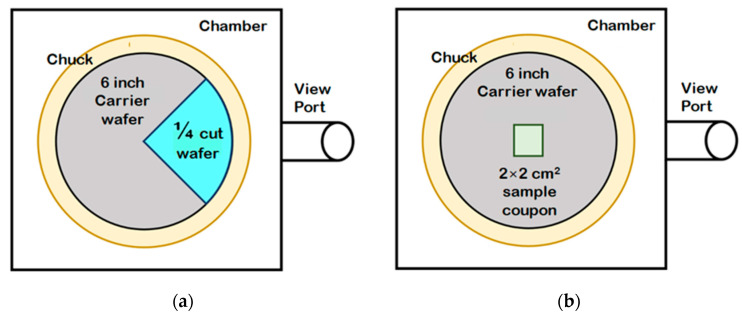
(**a**) Schematic illustration showing a wafer with one quarter coated by a single amorphous carbon layer and (**b**) the location of a coupon wafer on a multilayered specimen used to determine the ACL stripping endpoint.

**Figure 5:**
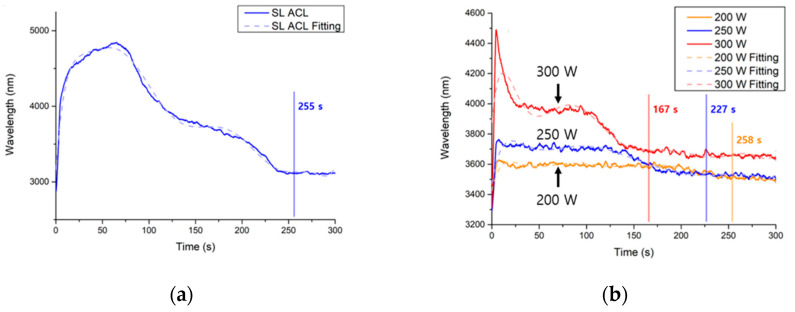
(**a**) OES spectrum acquired during stripping of a single amorphous carbon layer. Ninth-order polynomial fitting was performed to draw the regression line. The endpoint was detected at t = 255 s; (**b**) OES spectra of multilayered samples stripped at 200, 250, and 300 W with endpoints of 258 s, 227 s, and 167 s, respectively. Ninth-order polynomial fitting was performed to draw the regression lines.

Before analyzing the stripped specimens, we determined the stripping endpoints. One quarter of a single amorphous carbon layer on a Si wafer was analyzed to find an appropriate wavelength for EPD. This is illustrated schematically in Figure 4a. Multiple amorphous carbon layers were also deposited and stripped under the same conditions. Changes in intensity at 288 nm over time are shown in the OES spectrum in Figure 5a. We initially assumed that the CO signal would disappear at the stripping endpoint. After measuring ACL thickness several times and comparing the signal intensities at the selected wavelength, we found that the endpoint corresponded to a wavelength gradient of zero and an average intensity within a certain range.

We assessed multilayer stripping using our EPD algorithm. As shown in Figure 4b, the coupon wafer was located in the center of the carrier wafer. This made it difficult to detect the CO signal, because we could not focus the optical fiber on that location. We wondered whether methyl elimination from SiOC(H) would generate a noise signal that would indicate that damage was caused by plasma ion bombardment or sputtering. Exposure to the O_2_/Ar plasma transformed some methyl groups into CO, which ostensibly could be monitored at the same wavelength. However, changes in the signal at 288 nm in the spectra collected at 200, 250, and 300 W were similar, and the corresponding endpoints occurred at 258 s, 227 s, and 167 s, respectively, as shown in Figure 5b. These phenomena indicated that OES was not sensitive enough to detect noise resulting from methyl elimination. The plasma would thus damage the low-k films after stripping for 42 s, 73 s, and 133 s at 200 W, 250 W, and 300 W, respectively.

### 3.2. FT-IR Analysis

Depending on the Si–O bond angle, several peaks may appear in the FT-IR spectra of low-k thin films. Changes in the intensities of four peaks due to changes in bond structure, including Si–CH_3_ bonds, were monitored in the interval from 930 to 1330 cm^−1^ [14,15]. Si–O bonds with bond angles of less than 144° could be considered linear, and they generated a peak appearing at 1023 cm^−1^. The peak at 1063 cm^−1^ was indicative of a network structure with bond angles near 144°, while the peak at 1135 cm^−1^ reflected a cage structure with bond angles greater than 144° [14,15]. A peak attributed to Si–CH_3_ bonds appeared at 1273 cm^−1^, and peaks ascribed to C=C and C–O bonds in the ACLs were detected in the range from 1600 to 1700 cm^−1^ and at 1100 cm^−1^ [16].

**Figure 6:**
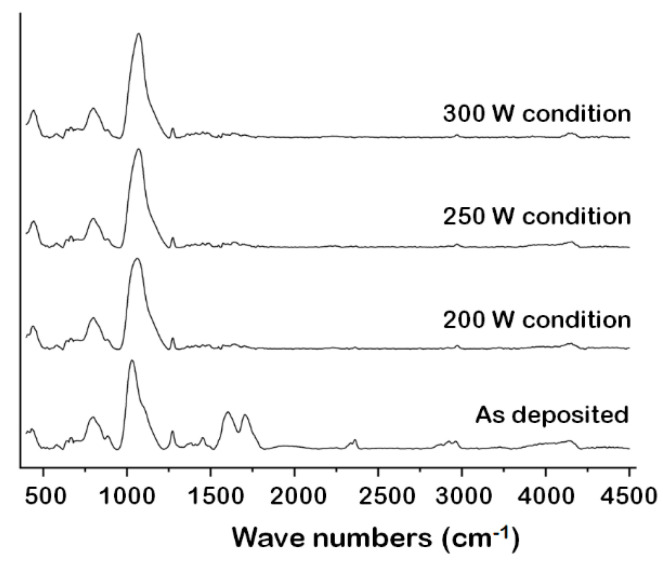
FT-IR spectra of a reference (as deposited) sample and samples stripped at 200, 250, and 300 W.

**Figure 7:**
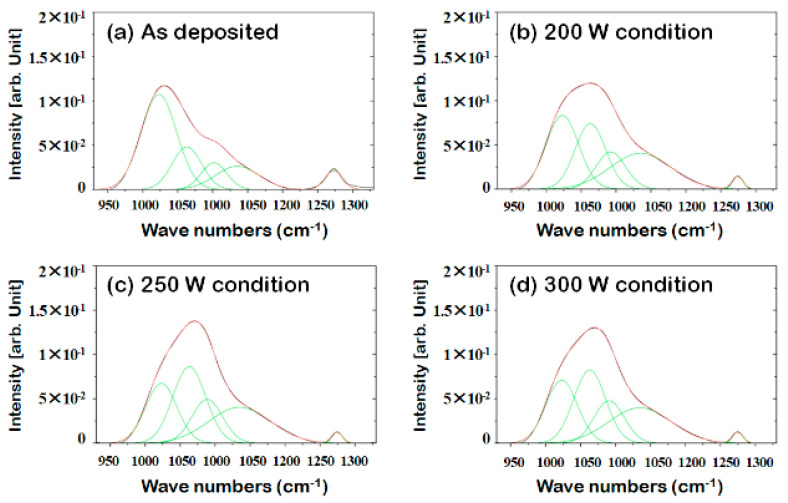
De-convoluted FT-IR spectra showing different Si–O peaks.

The Si–O peaks in the FT-IR spectra of a multilayered sample were monitored after separating the Si substrate, the SiOC(H) low-*k* dielectric, and the ACL hardmask, as shown in Figure 6. Most of the Si–O bonds were linear prior to stripping. Changes in the Si–O bond composition and a decrease in the number of Si–CH_3_ bonds were evident after stripping. The decrease in the number of linear Si–O bonds corresponded to an increase in the proportions of other structures. Plasma exposure during stripping also induced internal structural changes, such as the elimination of methyl groups. A peak attributed to C=C vibrations appeared at 1600 cm^−1^ after deposition, but it was not observed in the spectrum acquired after stripping. This confirmed that the ACL hardmask had been removed. The spectra in Figure 7 show the de-convoluted Si–O peaks after each process. 

**Figure 8:**
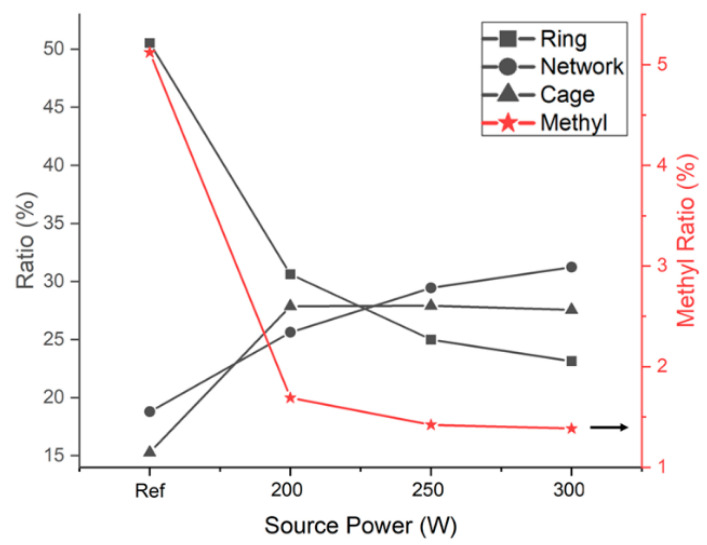
Percentages of carbon in rings, networks, cages, and methyl groups based on the deconvoluted FT-IR spectra.

Changes in the Si–O bond structure and the number of Si–CH_3_ bonds due to changes in the source power are illustrated in Figure 8. The network structure appeared to become more prevalent as the number of Si–CH_3_ bonds and linear structures decreased. Exposure to UV light emitted by the plasma and radial oxygen exposure can cause oxygen atoms in low-*k* films to diffuse [17]. Therefore, the increase in the proportion of network structures in the films may have been due to the removal of methyl radicals from linear structures. The removal of methyl groups or carbon and the formation of new bonds with Si or O atoms must be verified using additional analytical methods. Increasing the source power can increase the density of oxygen atoms in plasma [18]. Oxygen that diffuses into a film is reactive if ion bombardment does not occur [19]. This increases the oxygen concentration in the film and increases the probability of reactions with methyl groups within the film. Thus, an increase in the source power is accompanied by a decrease in the intensity of carbon peaks. This is also thought to be caused by additional plasma exposure after stripping.

### 3.3. XPS Analysis

**Figure 9:**
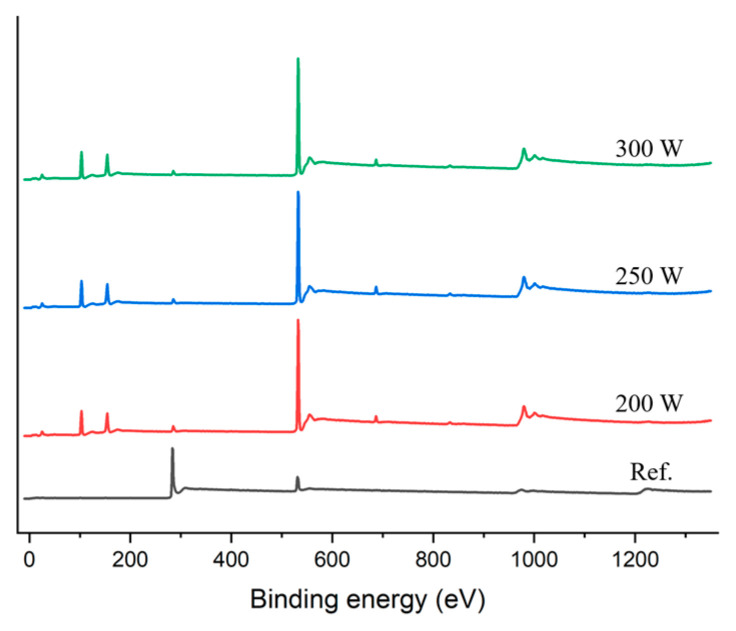
XPS survey spectra of a reference (as-deposited) sample and samples stripped at 200, 250, and 300 W.

**Figure 10:**
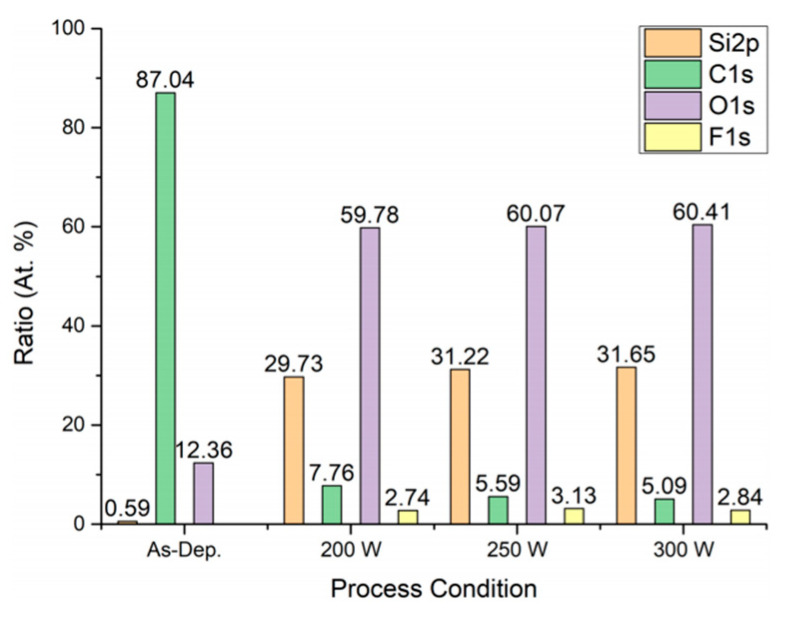
Atomic ratios based on XPS analysis.

**Figure 11:**
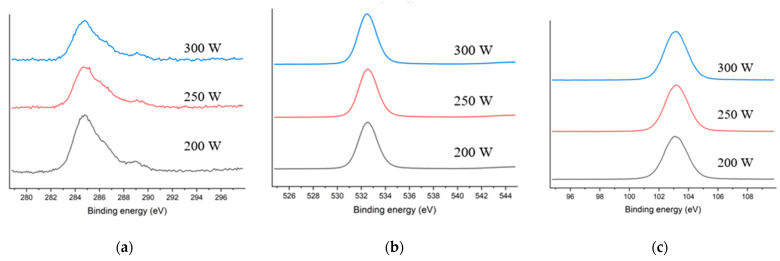
(**a**) C1s; (**b**) O1s and (**c**) Si2p spectra of samples stripped at 200 W, 250 W, and 300 W.

The XPS results for samples stripped at 200 W, 250 W, and 300 W are shown in Figure 9, Figure 10 and Figure 11. The atomic ratio of Si and O was approximately 1:2, which indicated the presence of a SiO_2_ skeleton structure. The proportion of carbon on the surface was 7.76 % after stripping at 200 W and 5.09 % after stripping at 300 W. This was consistent with a reduction in the number of Si–CH_3_ bonds as indicated by the FT-IR results. Therefore, the carbon peaks in the XPS spectra could be attributed to methyl groups within pores exposed on the surfaces of the films.

**Figure 12:**
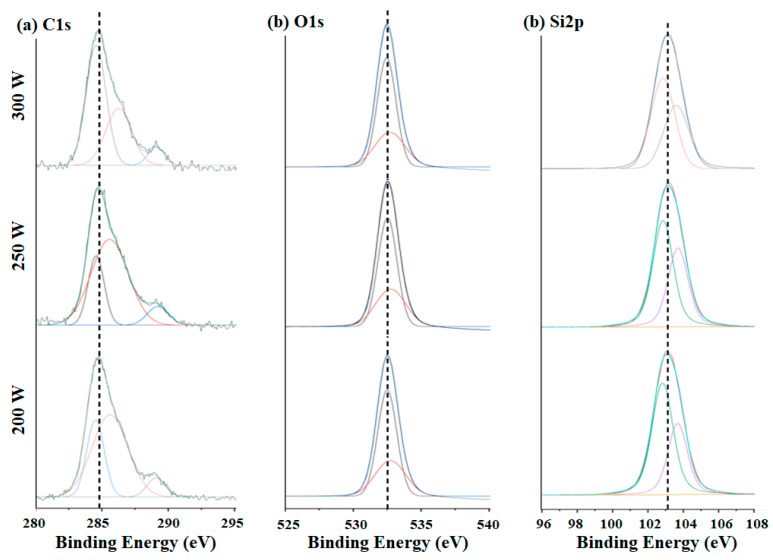
De-convoluted spectra of samples processed under different conditions: (**a**) C1s; (**b**) O1s; and (**c**) Si2p.

The de-convoluted C1s, O1s, and Si2p spectra are shown in Figure 12a–c, respectively. The C1s peak was de-convoluted into peaks that were ascribed to carbon in C≡O bonds, C–H bonds, and a-C. The O1s peaks were attributed to oxygen in C–O–C and Si–O–Si bonds. The proportions of O and C atoms that were bound to Si atoms could be determined by comparing the binding energies in the Si2p spectra of SiO_2_C_2_ and SiO_2_ or SiO_3_C [20,21]. Carbon in C–H bonds accounted for more than half of the peak intensity in the de-convoluted C1s spectra. The intensity of the peak attributed to oxygen in Si–O–Si bonds in the O1s spectra increased by approximately 4%, and the intensity of the Si2p peak was 5 % higher. This may have been due to the replacement of methyl groups or carbon atoms by Si atoms, which was consistent with the FT-IR results. Thus, the increase in the number of Si–O bonds on the surface could be attributed to a higher degree of network bonding.

### 3.4. Mechanical Properties

**Figure 13:**
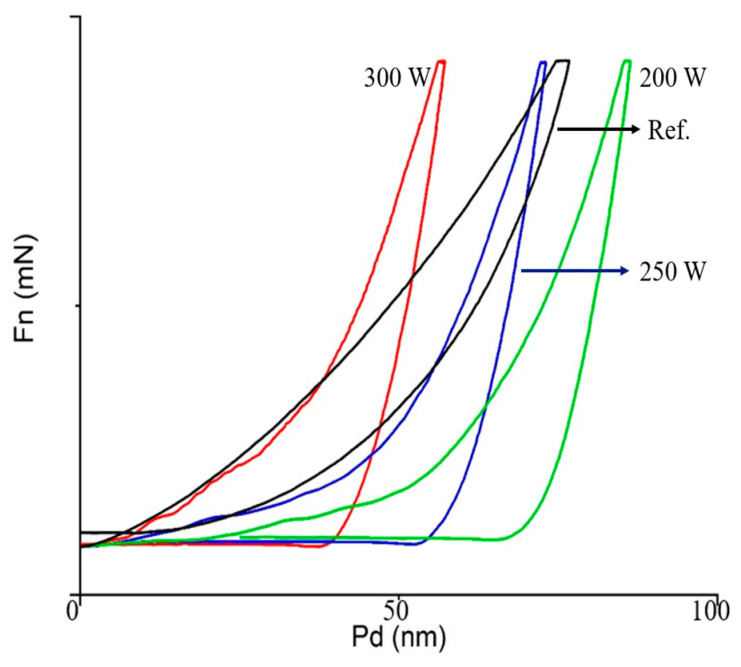
Nano-indentation results for an as-deposited sample and samples stripped at 200 W, 250 W, and 300 W.

**Figure 14:**
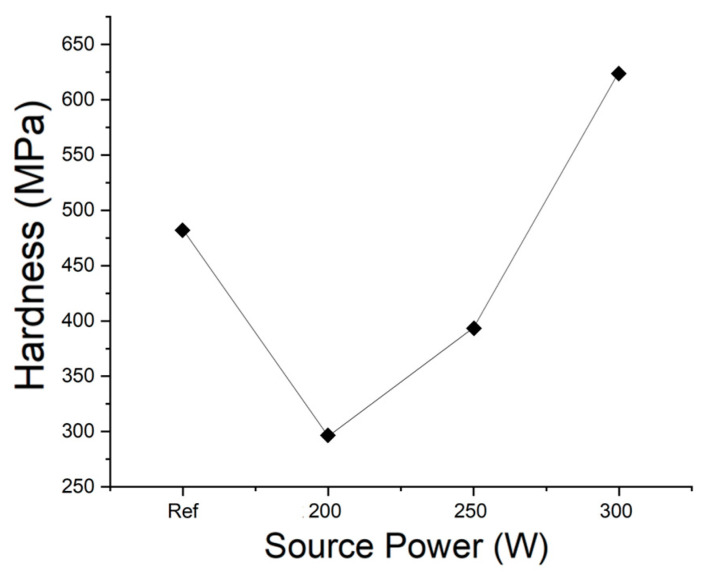
Hardness values of a reference (as-deposited) sample and samples stripped at 200 W, 250 W, and 300 W.

**Figure 15:**
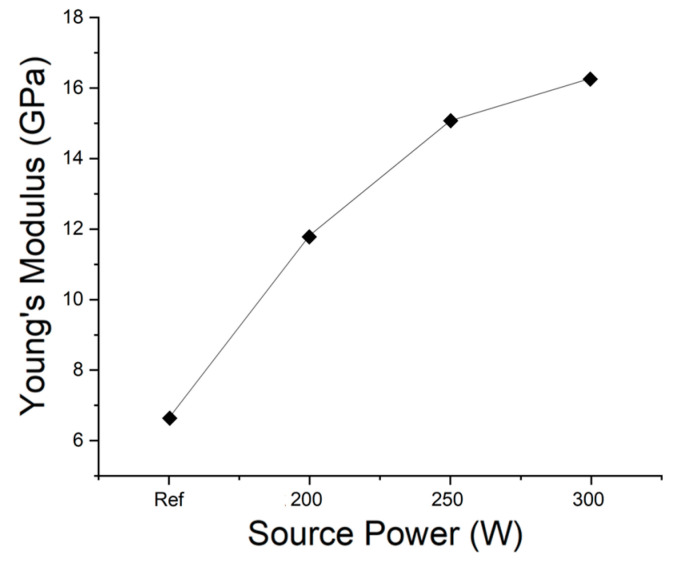
Young’s moduli of a reference (as-deposited) sample and samples stripped at 200 W, 250 W, and 300 W.

Mechanical strength was measured using a UNHT^3^ indentation tester (Anton Paar GmbH, Graz, Austria). The indentation results for a reference sample and samples subjected to stripping at 200 W, 250 W, and 300 W are shown in Figure 13. The hardness values and Young’s moduli of the samples are plotted in Figure 14 and Figure 15, respectively. The hardness of the thin films could be measured only after their surfaces were exposed by stripping. The hardness and modulus of the reference sample were those of the ACL surface prior to stripping. The hardness and modulus of the SiOC(H) thin film stripped at 200 W were approximately 0.3 and 11.8 GPa, respectively. The modulus was 16.2 GPa after stripping at 300 W, which represented an increase of ~37 %. Hardness and the Young’s moduli increased as the source power increased. A previous study determined [22]. The increase in Young’s modulus was due to an increase in the proportion of Si–O network bonds. Increasing the network structure and decreasing the suboxide structure can result in increasing mechanical strength, such as Young’s modulus [23]. In the results of FT-IR, we analyze the decrease in ring structure and the increase in network structure. Compared to previous studies, the higher the amount of energy applied to the thin film in experimental conditions that increase to 200–300 W, the greater the change in the mechanical properties of the thin film. Therefore, changes in chemical bonding indicated that plasma exposure caused internal structural changes, which confirmed that plasma exposure could alter the physical properties of the films.

## 4. Conclusions

In this study, SiOC(H) low-*k* dielectric thin films on silicon substrates were coated with amorphous carbon masks, and the effects of ACL stripping on the underlying films were evaluated. Optical information about the plasma was collected via OES during the ACL stripping process. FT-IR analysis, XPS analysis, and indentation measurements were also performed to characterize the thin films. The FT-IR results showed that the number of Si–O network bonds was higher after stripping the films, and the XPS results indicated that stripping at 200 W increased the number of Si–O–Si bonds by 5%. Increasing the source power applied during stripping resulted in a higher degree of network bonding. This was indicated by an approximately 37% increase in Young’s modulus. These changes indicated that the internal chemical structures of the SiOC(H) films could be altered by subsequent processes, such as plasma exposure during ACL stripping. Changes in the properties of SiOC(H) thin films caused by such processes should be considered when devices are designed. Therefore, in-situ endpoint detection to diagnose sensors should be performed using a technique, like OES. Since SiCO(H) thin films are used as an ILDs, their electrical properties must also be analyzed. Since heat can alter their characteristics, as well, changes induced by heat treatment, along with changes in the electrical properties of SiOC(H) thin films, should be evaluated in follow-up studies. The limitation of the proposed research is the measurement of the dielectric constant of the TMCTS-based SiCOH to investigate the effect of RF power, as well as other process parameters, and the raised limitation of this research will be continued for up-coming research objective. 

## Data Availability

Data is available upon the request.

## References

[1] (2019). IEEE International Roadmap for Device and System 2018 Edition.

[2] Cheng Y.-L., Lee C.-Y., Kandelousi M.S. (2018). Porous Low-Dielectric-Constant Material for Semiconductor Microelectronics. Nanofluid Flow in Porous Media.

[3] Jung I.-S., Hong S. (2018). Characterization of Plasma Deposited TMCTS Based low-*k* Thin Film Deposition Process. Sci. Adv. Mater..

[4] Bilodeau S.M., Borovik A.S., Ebbing A.A., Vestyck D.J., Xu C., Roeder J.F., Baum T.H. (2004). Chemical Routes to Improved Mechanical Properties of PECVD Low K Thin Film. MRS Online Proceedings Library.

[5] Miyajima H., Ishikawa K., Sekine M., Hori M. (2019). Review of Methods for the Mitigation of Plasma-Induced Damage to Low-Dielectric-Constant Interlayer Dielectrics Used for Semiconductor Logic Device Interconnects. Plasma Process. Polym..

[6] Lopaev D.V., Zyryanov S.M., Zotovich A.I., Rakhimova T.V., Mankelevich Y.A., Voronia E.N. (2020). Damage to Porous SiOCH low-*k* Dielectrics by O, N and F atoms at lowered temperatures. J. Phys. D Appl. Phys..

[7] Jang J.K., Tak H.W., Yang K.C., Shin Y.J., Hyeon J.Y., Hyeon J.Y., Kang M.G., Ahn J.H., Yeom G.Y. (2019). Etch Damage Reduction of Ultra low-*k* Dielectric by Using Pulsed Plasmas. ESC Trans..

[8] Torazawa N., Matsumoto S., Harada T., Molinearaga Y., Inagaki D., Kabe T., Hirao S., Seo K., Suzuki S., Korogi H. (2016). High-Performance Extremely low-*k* Film Integration Technology with Metal Hardmask Process for Cu Interconnects. ESC J. Solid State Sci. Technol..

[9] Lee D., Tatti P., Lee R., Chang J., Cho W., Bae S. Study for New Hardmask Process Scheme. Proceedings of the Advanced in Patterning Materials and Processes XXXIV.

[10] Payne M., Lippy S., Lieten R., Kesters E., Le Q.T., Murdoch G., Gonzalez V.V., Holsteyns F. (2016). Evaluation of Post Etch Residue Cleaning Solution for the Removal of Tin Hardmask after Dry Etch of low-*k* Dielectric Materials on 45 nm Pitch Interconnects. Solid State Phenom..

[11] Lai C.C., Chang Y.H., Chien H.J., Lu M.C. Amorphous Carbon Process Optimization to Increase Hardmask and Lithographic Capabilities by Its Step Coverage Improvement. Proceedings of the 6th International Symposium on Next Generation Electronics.

[12] Mankelevich Y.A., Voronina E.N., Rakhimova T.V., Palov A.P., Lopaev D.V., Zyryanov S.M., Baklanov M.R. (2016). Multi-step Reaction Mechanism for F atom interactions with organosilicate glass and SiOx films. J. Phys. D Appl. Phys..

[13] Kim K.P., Song W.S., Park M.K., Hong S. (2021). Surface Analysis of Amorphous Carbon Thin Film for Etch Hardmask. J. Nanotechnol. Nanomater..

[14] Grill A., Neumayer D.A. (2003). Structure of low dielectric constant to extreme low dielectric constant SiCOH films: Fourier transform infrared spectroscopy characterization. J. Appl. Phys..

[15] Baklanov M.R., de Marneffe J.-F., Shamiryan D., Urbanowicz A.M., Shi H., Rakhimova T.V., Huang H., Ho P.S. (2013). Plasma processing of low-*k* dielectrics. J. Appl. Phys..

[16] Ţucureanu V., Matei A., Avram A.M. (2016). FTIR Spectroscopy for Carbon Family Study. Crit. Rev. Anal. Chem..

[17] Li G., Zheng G., Ding Z., Shi L., Li J., Chen Z., Wang L., Tay A.A.O., Zhu W. (2018). High-performance ultra-low-*k* fluorine-doped nanoporous organosilica films for inter-layer dielectric. J. Mater. Sci..

[18] Kitajima T., Noro K., Nakano T., Makabe T. (2004). Influence of driving frequency on oxygen atom density in O2radio frequency capacitively coupled plasma. J. Phys. D Appl. Phys..

[19] Takeda K., Miyawaki Y., Takashima S., Fukasawa M., Oshima K., Nagahata K., Tatsumi T., Hori M. (2011). Mechanism of plasma-induced damage to low-*k* SiOCH films during plasma ashing of organic resists. J. Appl. Phys..

[20] Hamdan A., Abdul Halim R., Anjum D., Cha M.S. (2017). Synthesis of SiOC:H nanoparticles by electrical discharge in hexamethyldisilazane and water. Plasma Process. Polym..

[21] Meškinis Š., Vasiliauskas A., Andrulevičius M., Peckus D., Tamulevičius S., Viskontas K. (2020). Diamond Like Carbon Films Containing Si: Structure and Nonlinear Optical Properties. Materials.

[22] Gourhant O., Gerbaud G., Zenasni A., Favennec L., Gonon P., Jousseaume V. (2010). Crosslinking of porous SiOCH films involving Si–O–C bonds: Impact of deposition and curing. J. Appl. Phys..

[23] Huang C.H., Huang H.L., Hung C.I., Wang N.F., Wang Y.H., Houng M.P. (2008). Bond Structure in Porous SiOCH low-*k* Film Fabricated by Ultraviolet Irradiation. Jpn. J. Appl.Phys..

